# High Curie Temperature Achieved in the Ferromagnetic Mn_x_Ge_1−x_/Si Quantum Dots Grown by Ion Beam Co-Sputtering

**DOI:** 10.3390/nano12040716

**Published:** 2022-02-21

**Authors:** Xiaoxiao Duan, Shuming Ye, Jing Yang, Chen Li, Chunjiang Lu, Xinpeng He, Luran Zhang, Rongfei Wang, Feng Qiu, Jie Yang, Haoyang Cui, Chong Wang

**Affiliations:** 1 National Center for International Research on Photoelectric and Energy Materials, School of Materials and Energy, Yunnan University, Kunming 650091, China; duanxiaoxiao10@163.com (X.D.); ysming97@163.com (S.Y.); li18281543317@163.com (C.L.); chunjiangtenet@163.com (C.L.); valvrave@126.com (X.H.); zhangluran@ynu.edu.cn (L.Z.); fengqiu@ynu.edu.cn (F.Q.); 2 Key Lab of Polar Materials and Devices, Ministry of Education, East China Normal University, Shanghai 200241, China; jyang@ee.ecnu.edu.cn; 3 College of Electronic and Information Engineering, Shanghai University of Electric Power, Shanghai 200090, China; cuihy@shiep.edu.cn

**Keywords:** MnGe quantum dots, ferromagnetic nanostructure, Curie temperature, doping level

## Abstract

Ferromagnetic semiconductors (FMSs) exhibit great potential in spintronic applications. It is believed that a revolution of microelectronic techniques can take off, once the challenges of FMSs in both the room-temperature stability of the ferromagnetic phase and the compatibility with Si-based technology are overcome. In this article, the Mn_x_Ge_1−x_/Si quantum dots (QDs) with the Curie temperature (*T_C_*) higher than the room temperature were grown by ion beam co-sputtering (IBCS). With the Mn doping level increasing, the ripening growth of MnGe QDs occurs due to self-assembly via the Stranski–Krastanov (SK) growth mode. The surface-enhanced Raman scattering effect of Mn sites observed in MnGe QDs are used to reveal the distribution behavior of Mn atoms in QDs and the Si buffer layer. The Curie temperature of Mn_x_Ge_1−x_ QDs increases, then slightly decreases with increasing the Mn doping level, and reaches its maximum value of 321 K at the doping level of 0.068. After a low-temperature and short-time annealing, the *T_C_* value of Mn_0.068_Ge_0.932_ QDs increases from 321 K to 383 K. The higher Ge composition and residual strain in the IBCS grown Mn_x_Ge_1−x_ QDs are proposed to be responsible for maintaining the ferromagnetic phase above room temperature.

## 1. Introduction

In the last two decades, massive efforts have been devoted to probing one kind of multifunctional material, on which the so-called three golden disciplines, namely, optics, microelectronics, and magnetism, can be integrated organically, so that the optical, electronic, and magnetic responses can be modulated effectively in one kind of material by the external fields [1,2]. If it actualizes a new information technology, the spin electron substitutes for the charge as the information carrier, characterized by ultrafast transportation, high-capacity, ultra-wideband, and ultralow-power dissipation [1,3,4]. In that case, it is worthy of expecting such results in these kinds of materials. This technology can overcome many predicaments of the traditional charge electronics, since the interaction energy between spins is 1000 times less than that between charged electrons. In other words, changing the former states is much easier than that of the latter [5]. Diluted ferromagnetic semiconductors (DFMS) have been regarded as one of the most promising candidate materials for fulfilling this excellent integration engineering. Compared to the II-VI, III-V compounds, and halide perovskite counterparts [6], group-IV DFMSs have continued to attract more attention due to their natural advantages of direct compatibility with Si-based microelectronic techniques and the higher Curie temperature (*T_C_*) observed in the previous work [4,7].

A *T_C_* value much higher than room temperature (*T_C_* > 450 K) is the prerequisite for spintronic application. Therefore, in recent years, there have been constant efforts to break the fundamental limitations of the *Tc.* Park et al. grew the Mn_x_Ge_1−x_ ferromagnetic thin films on the GaAs substrate using the molecular beam epitaxy (MBE) technique [7]. The *T_C_* value of these films increases linearly from 25 K to 116 K, with the Mn doping level increasing from 0.006 to 0.035. The relatively low solid solubility of Mn in the Ge matrix prevents the Mn_x_Ge_1−x_ alloy from obtaining a higher *T_C_*. Many previous studies have shown that intermetallic compounds precipitate in Mn_x_Ge_1−x_ thin films with a high Mn doping level, such as Mn_5_Ge_3_ (*T_C_*~296 K) and Mn_11_Ge_8_ (*T_C_*~270 K) [8,9,10], for instance, with a typical Mn doping level higher than 0.05 [11]. Xiu et al. fabricated the pure-phase Mn_0.05_Ge_0.95_/Si quantum dots (QDs) [11,12]. Their *T_C_* values, and the ferromagnetic temperature (FMT) manipulated by a static electric field were promoted to 400 and 100 K, respectively. The high-*T_C_* above 400 K achieved in MnGe QDs can probably be ascribed to at least two apparent advantages of the low dimensional nanostructure. First, the formation of metal precipitate phases is restrained in the low dimensional DFMS; this behavior increases the ratio of the doping Mn acting as the substitution atom and the localized Mn moment, rather than the interstitial one [13]. The other is that the generation of quantum-confined effects enhances the carrier localization and the exchange coupling between carriers and the localized Mn moment. Furthermore, the low dimensional DFMS with a *T_C_* value above room temperature has also been demonstrated recently in other nanostructures, such as nanowires and nanotubes [14,15]. Although these achievements were accessed for the commercial application of the DFMSs, the current controllable FMT is still too low and should further improve at room temperature [16]. However, the systematically manipulating mechanisms of many common factors, such as Mn doping level, residual stress, and post-annealing, etc., on the ferromagnetism and the *T_C_* value of the low dimensional MnGe DFMS are still absent. Additionally, almost all previously reported MnGe QDs, which are fabricated using the refined MBE technique, are usually deemed to have a relatively high cost and complicated processing method, and are not readily compatible with the so-called self-aligned silicide process [17].

Ion beam sputtering deposition (IBSD) is crucial for preparing high-quality thin films, demonstrating that high-quality epitaxial thin films and low dimensional nanomaterials could actualize through IBSD [17,18,19,20,21]. More importantly, the stress and relaxation in these lattice-mismatched nanostructures can be well-tuned by IBSD [22]. In this article, the IBSD technology used to grow the Mn_x_Ge_1−x_ QDs, and the growth mechanism and Mn-doping-level dependent ferromagnetic evolution of these DFMS QDs is addressed well.

## 2. Experimental Section

**Figure 1:**
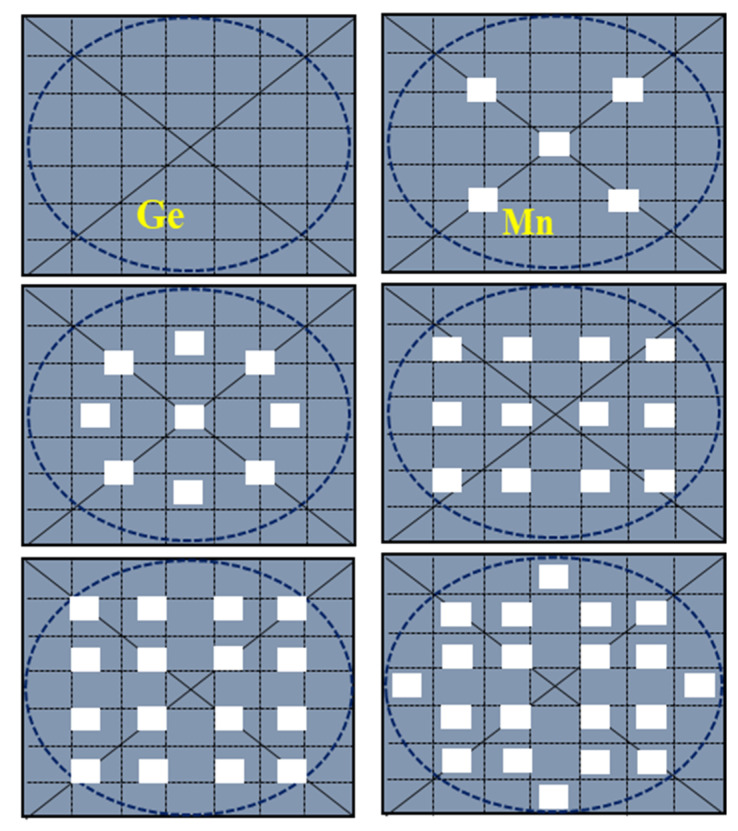
The patterns of Mn slices distributed on Ge target with an increase in Mn-slice number.

Ge-Mn composite target can be manufactured by pasting a certain amount of high purity Mn slices, each with a size of 5 mm × 5 mm × 0.5 mm, on a single crystal Ge target (99.999% purity, size of 70 mm × 70 mm). Figure 1 shows the several patterns of Mn slices distributed on a Ge target to increase the Mn doping level gradually in MnGe QDs. These slices are limited to a circle with a radius of ~3.53 cm that corresponds to the largest bombardment area of the Ar^+^ ion beam.

The samples were grown on n-Si (001) substrate by using ion beam co-sputtering techniques. The Kaufman-type ion source (caliber: 30 mm) was used to generate an Ar^+^ ion beam [23]. The background vacuum and depositing Ar-gas pressure were 2.3 × 10^−6^ Torr and 2.7 × 10^−4^ Torr, respectively. The Si substrate was cleaned, first by using a modified Shiraki procedure [24]; then, the substrate was rinsed with 2.5% hydrofluoric acid (HF) to remove the natural oxide layer on the surface to obtain a hydrogen passivation surface. Finally, the substrate is dried with high-purity nitrogen and placed in a vacuum chamber.

A 50 nm thick Si buffer layer was deposited at 750 °C before growing the Mn_x_Ge_1−x_ QDs layer. The current settings for sputtering Si and Ge-Mn composite targets were 10 and 7 mA. As a result, the deposition rates of the Si buffer layer and the Mn_x_Ge_1−x_ QDs layer, monitored by a quartz crystal oscillator, were 0.022 and 0.011 nm/s, respectively. The growth temperature of the Mn_x_Ge_1−x_ QDs layer was set to 700 °C for all samples with different Mn concentrations, and the layer was deposited with a nominal thickness of about 3 nm. Immediately after depositing the Mn_x_Ge_1−x_ layer, the samples were cooled to room temperature. Rapid thermal processing is used for post-annealing in a pure N_2_ environment at a temperature of 465 °C for 20 min.

The components of the sample surface were determined by using an energy dispersive X-ray spectrometer (EDX) equipped with a scanning electron microscopy (SEM) system. The surface morphologies of samples were measured ex situ by an atomic force microscope (AFM) in tapping mode. Raman scattering spectra were conducted with excitation wavelengths of 514.5 nm in the back-scattering geometry at room temperature. X-ray photoelectron spectroscopy analyses were carried out using a Thermo Scientific K-Alpha XPS system with a monochromatic Al Kα X-ray source. Magnetic measurements were performed in the Physical Property Measurement System (PPMS-9). The applied external magnetic field is parallel to the surface of the sample. The diamagnetic contribution from the substrate has been subtracted.

## 3. Results and Discussion

**Figure 2:**
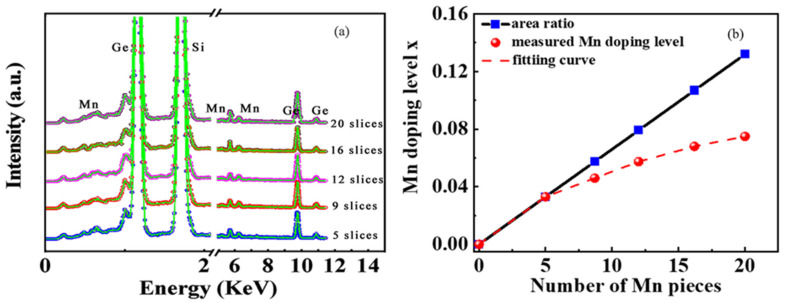
(**a**) The X-ray energy spectrum of the samples with an increase in Mn-slice number. (**b**) The Mn-slice number dependence of the Mn content x in the Mn_x_Ge_1−x_ film grown by ion beam co-sputtering.

The composite Ge-Mn target can be manufactured by pasting a certain amount of high purity Mn slices onto a single crystal Ge target and the Mn doping level x in the Mn_x_Ge_1−x_ QDs, as a function of the Mn slice number on the Ge target, can be construed. With the Mn-slice number increasing from 5 to 20, the actual Mn doping levels of 0.032, 0.046, 0.058, 0.068, and 0.075 can be determined by the X-ray energy spectrum, respectively, as shown in Figure 2a. Mn content in the MnGe QDs does not increase linearly with the Mn-slice number, but can be fitted well by an exponential function in Figure 2b. This dependence starts to diverge from the nominal values based on the area ratio of all Mn-slices to Ge-target when the Mn-slice number is more than five. The main reason for this deviation is that the maximum coverage area of ion beam sputtering is a circular area with a radius of 3.5 cm. Since the sputtering area is not easy to control precisely, the actual sputtering area will be smaller than the theoretical area of the target.

**Figure 3:**
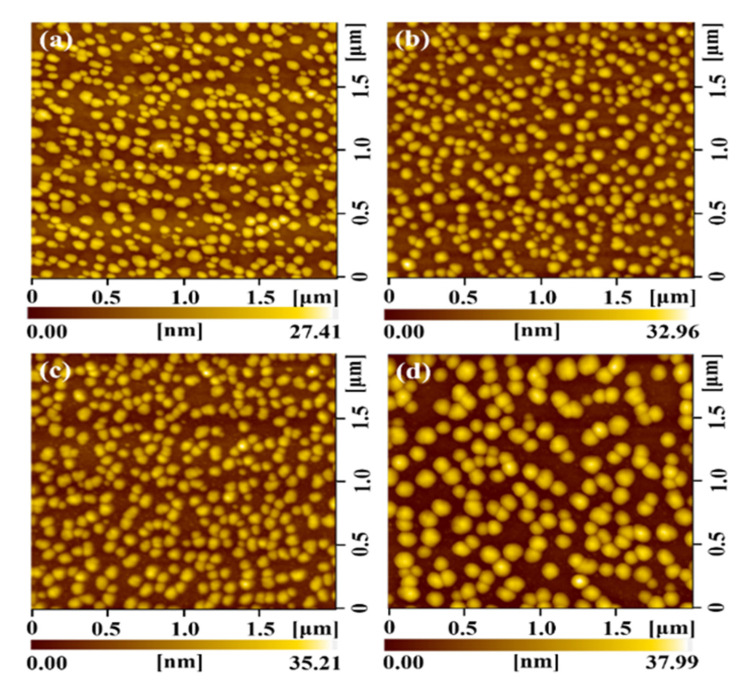
AFM surface morphology images of MnGe QD samples with Mn content of 4.6% (**a**), 5.8% (**b**), 6.8% (**c**) and 7.5% (**d**), respectively.

The epitaxial growth mechanism of MnGe/Si QDs is close to that of Ge/Si QDs [11,12,16], and is based on the stress relaxation process originating from the lattice mismatch between Si and Ge. In the process, these QDs can be formed on the Si buffer layer by self-assembly via the Stranski–Krastanov (SK) growth mode, proving that IBSD is also an epitaxial growth technology [18]. Therefore, the growth of Ge/Si QDs by IBSD has been studied in detail [20,22,25]. In our work, MnGe/Si QDs with different Mn doping levels are prepared by IBSD. Figure 3 shows self-assembled MnGe QDs randomly distributed on Si substrate. The density of MnGe QDs decreases from 1.4 × 10^10^ cm^−2^ to 6.1 × 10^9^ cm^−2^, and the MnGe QDs size increases with the Mn content. These evolutions agreed with that of their counterparts grown by MBE [10]. Furthermore, similar density-decrease and size-increase behaviors found in the pure Ge QDs, which experienced the post-annealing process, were attributed to the diffusion of Ge adatom and the Ostwald ripening growth of Ge islands [26,27]. It suggested that the bonding amount of Mn-Ge during the initial stage of nucleation formation can enhance the diffusion of Ge adatom and favor ripening growth.

**Figure 4:**
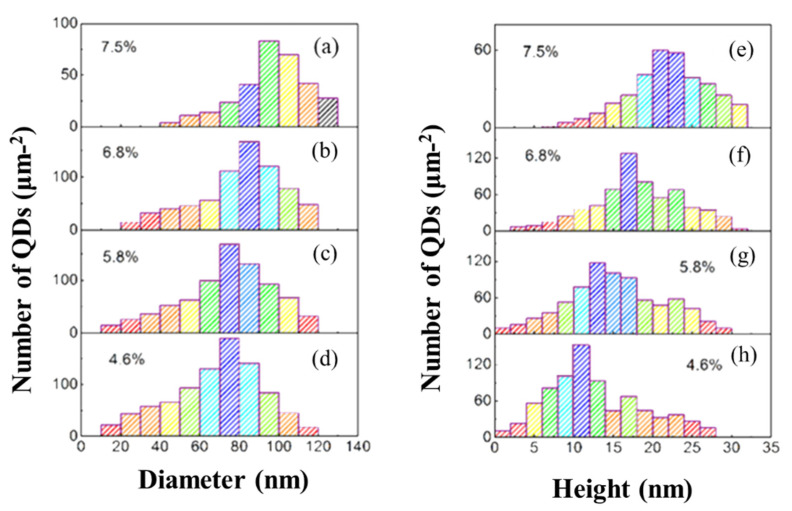
Statistic histograms for the diameter (**a**–**d**) and height (**e**–**h**) of the MnGe QDs at different Mn contents.

As shown in the statistical histograms in Figure 4, as the Mn doping increases from 0.046 to 0.075, both the average diameter and height of the Mn_1−x_Ge_x_ QDs increase, from 75 nm to 100 nm and from 11 nm to 23 nm, respectively. The relatively drastic increase in the height of QDs probably can be profiled by comparing the average aspect ratio of these Mn_1__−x_Ge_x_ QDs. Yet, the number of small quantum dots decreased, while the large quantum dots appeared and increased. These phenomena suggest that the evolution mechanism of Mn_1−x_Ge_x_ QDs follows the Ostwald ripening process with increasing Mn doping level [28].

Usually, the evolution of QDs’ size is accompanied by the variation in QDs’ morphology [29]. According to the island shape’s dependence on the contact-angle, the shape of Ge/Si QDs with a contact angle *θ* less than 7° and larger than 13° can be classified as pre-pyramid and dome [30], respectively, and the rest of the Ge/Si QDs are pyramid or hut. Increasing the Mn doping level from 0.046 to 0.075, the ratio of dome islands improved from 94% to 99%. It is proposed that the ferromagnetism of MnGe QDs originates primarily from the large dome-shaped MnGe QDs rather than the small hut-shaped ones [16]. Thus, the high ratio of dome islands realized in this work implies the stronger ferromagnetism of MnGe QDs.

**Figure 5:**
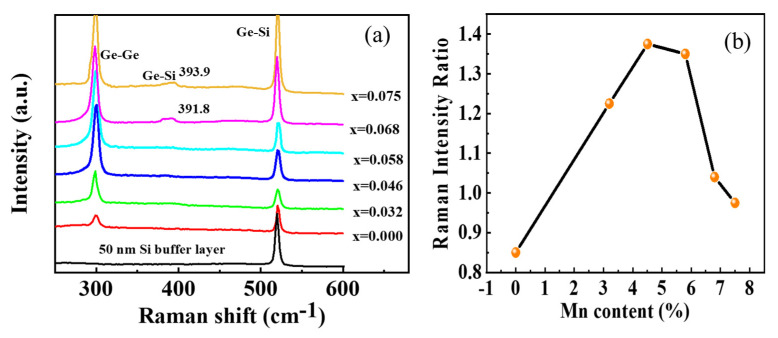
(**a**) Raman spectra of the MnGe QDs samples with different Mn contents and the sample with only Si buffer layer. (**b**) Raman intensity ratio of Ge-Ge peak to Si-Si peak dependence of Mn concentration.

As shown in Figure 5a, the sharp Raman peak at ~520 cm^−1^ originates from the Si-Si bond vibration of the Si buffer layer, indicating the high crystallinity of the Si buffer layer. The Raman shift of crystalline Ge peak at ~300 cm^−1^ does not change obviously with the variation in the Mn doping level, while the intensity ratio of Ge-Ge to Si-Si Raman peak varies significantly, as shown in Figure 5b. Actually, it first increases, then decreases with the increase in Mn doping level, and reaches its maximum of 1.37 in the Mn_0.046_Ge_0.954_ QDs. It is interesting to note that the scattering intensity of Ge-Ge peak (*I*_Ge-Ge_) is stronger than that of Si-Si one (*I*_Si-Si_) in most of the Mn_x_Ge_1−x_ QDs, except for those without doping and those with the maximal doping level (x = 0.075). Generally, the relative intensity change in Raman peaks is proportional to the variation in a relative number of corresponding bonds in films [27]. In this experiment, the number of Ge-Ge bonds in the MnGe QDs samples decreases monotonically with the increase in Mn content, since the deposition time is the same for all QDs samples. However, the relative *I*_Ge-Ge_ value does not decrease correspondingly in the MnGe QDs samples, suggesting another factor dominates the variation in these Raman peaks.

Surface-enhanced Raman spectroscopy (SERS) has been widely investigated by cladding Ag and Au films onto the surface of all kinds of samples, but little work is concerned with the transition metal coating. Recently, some organic films have demonstrated the SERS behaviors, including the Mn nanostructures [31]. This suggests that the abnormal Raman peak intensity observed in MnGe/Si QDs can be attributed to the surface-enhanced Raman scattering from Mn sites. Within this frame, the enhancement of surface plasmons in the MnGe layer is proposed to result in the increase in relative *I*_Ge-Ge_ values in the Mn doping level range from 0 to 0.046. However, the intensity ratio of *I*_Ge-Ge_/*I*_Si-Si_ slightly decreases to 1.35 for the Mn_0.058_Ge_0.942_ QDs, which is associated with the combined effect of the two factors. First, the enhanced Raman scattering of Ge-Ge bonds from surface plasmons can be weakened by the continuous reduction in the Ge-Ge bond amount as the Mn doping level reaches a relatively high level. Second, some Mn atoms can also diffuse into the Si buffer layer during the island growth and induce the Raman signal from Si-Si bonds to be enhanced by the surface plasmons simultaneously. The higher the Mn doping level, the more the Mn atoms diffuse into the buffer layer, and the stronger the enhanced intensity of Si-Si peak from surface plasmons. Both factors can lead to the continuous decrease in the *I*_Ge-Ge_/*I*_Si-Si_ ratio. Once the Mn doping level is higher than 0.058, the Raman peak of Si-Ge bonds appears at ~391 cm^−1^, which indicates that the Si-Ge intermixing occurring in the islands results from the Ostwald ripening growth. Since the Ge content in Ge/Si islands can be calculated according to the Raman shifts of Ge-Ge and Si-Ge peaks [32,33], 90.9% and 89.1% in the Mn_0.068_Ge_0.932_ and Mn_0.075_Ge_0.925_ QDs, is determined by a similar method in which the Mn-doping related effects are neglected, only the Ge-Si intermixing is taken into account. Generally, the Ge-Si intermixing is inevitable in the Ge/Si QDs during the physical growth processes. This higher Ge composition in MnGe QDs is beneficial for increasing their Curie temperature [16]. Thus, it is worth noticing that the Ge chemical composition in the IBSD-grown Ge/Si QDs is higher than that in MBE-grown QDs [32,33], and a better ferromagnetism for MnGe QDs could expected by using IBSD. For the Mn_0.075_Ge_0.925_ QDs, the relative intensity *I*_Si-Si_ could further increase, even exceeding the *I*_Ge-Ge_ again. The depletion of Ge-Ge bonds via Ge-Si intermixing should also be considered at this Mn doping level.

**Figure 6:**
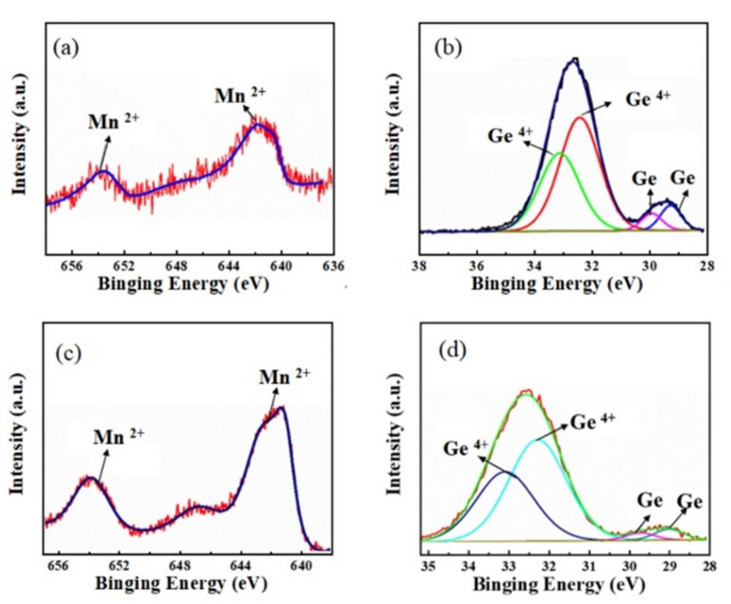
XPS spectra and their fitted lines for Mn_0.068_Ge_0.932_ (**a**,**b**) and Mn_0.075_Ge_0.925_ (**c**,**d**) QDs sample.

To probe the role of Mn ions, we also characterized the chemical binding states of Mn_0.068_Ge_0.932_ and Mn_0.075_Ge_0.925_ QDs samples using the X-ray photoelectron spectroscopy (XPS) technique, as shown in Figure 6. In the former sample, the binding energies of Mn 2p_3/2_ and Mn 2p_1/2_ states are located at 641.4 eV and 653.3 eV, respectively, indicating the two valent states of the Mn ion [34,35,36]. The binding energies of the two states undergo a shift of −0.30 eV and 0.50 eV in the latter sample, respectively. The blueshift of binding energy suggests that a higher valence state of Mn exists in the Mn_0.075_Ge_0.925_ QDs sample [36]. Moreover, for the Mn_0.068_Ge_0.932_ QDs, the peaks of Ge 3d_5/2_ and Ge 3d_3/2_ located at 29.32 eV and 29.82 eV can be ascribed to the zero valences of Ge, while those peaks located at 32.44 eV and 32.75 eV match with the +4 valence of Ge, respectively. With the doping level increasing from 0.068 to 0.075, the binding energy of zero-valent Ge shifts 0.15 eV and 0.30 eV, respectively, which implies the formation of the higher Ge valence states [37,38,39]. The coexistence of the higher valence states of both Mn and Ge in the Mn_0.075_Ge_0.925_ QDs sample results from the formation of the second phase Mn-Ge structure [34].

Therefore, the atomic structure of MnGe QDs has changed with the increase in the Mn doping level in the MnGe QDs samples. At a low doping level, the doping Mn atoms take the substitutional sites, and the crystal structure of QDs retains its diamond structure. However, at a high doping level, a new crystal structure accompanied by the possible formation of the intermetallic precipitate, such as Mn_5_Ge_3_ with its lattice symmetry of *P6_3_/mcm* [40], may also be produced and coexist with the diamond structure in the MnGe islands. Combined with the observation of AFM, Raman, and XPS, the composition and structure transition of QDs mainly occurs at the interface between the island bottom and the Si buffer, from the MnGe QDs, MnGeSi QDs, to MnGeSi QDs accompanied by a small quantity of precipitate, with an increase in the Mn doping level.

**Figure 7:**
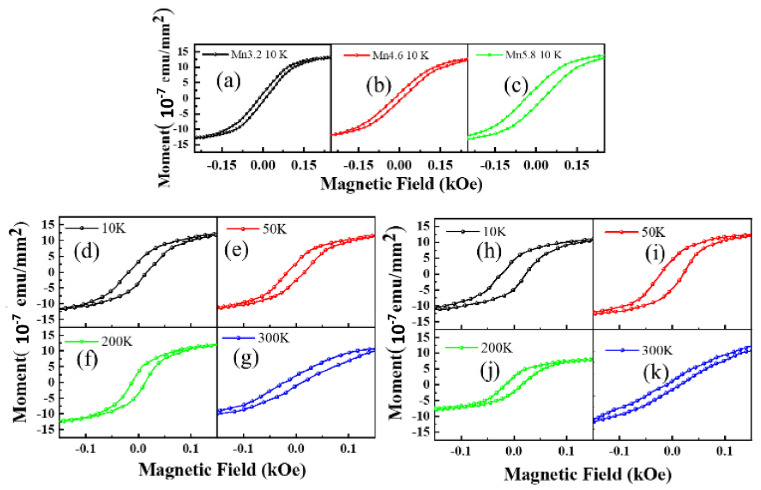
The hysteresis loops of samples with different Mn dopant concentrations at different test temperatures. The hysteresis loops of Mn_0.032_Ge_0.968_, Mn_0.046_Ge_0.954_ and Mn_0.058_Ge_0.942_ QDs samples at 10 K (**a**–**c**) and the hysteresis loops of Mn_0.068_Ge_0.932_ (**d**–**g**) and Mn_0.075_Ge_0.925_ QDs (**h**–**k**) samples at different temperatures from 10 K to 300 K, respectively. It is noted that the diamagnetic contributions from the substrates were subtracted.

The magnetic properties of Mn_x_Ge_1−x_/Si QDs were characterized by the PPMS-9. The applied external magnetic field is parallel to the surface of the sample. Figure 7 shows the hysteresis loops of these QDs samples with different Mn doping levels, in which the diamagnetic contribution from the substrate has been subtracted. The remanence and coercive force of samples increases with the Mn doping concentration increasing from 0.032 to 0.058 (see Figure 7a), which indicates that the Curie temperature of Mn_x_Ge_1−x_ QDs can be promoted effectively by simply increasing the Mn doping level. In addition, the hysteresis loops of Mn_0.068_Ge_0.932_ and Mn_0.075_Ge_0.925_ QDs were recorded at different temperatures, as shown in Figure 7d–k). At 300 K, the typical remanences of 1.25 × 10^−7^ emu/mm^2^ and 0.36 × 10^−7^ emu/mm^2^ can still be observed in Mn_0.068_Ge_0.932_ and Mn_0.075_Ge_0.925_ QDs, respectively, which suggests high *T_C_* above room temperature.

**Figure 8:**
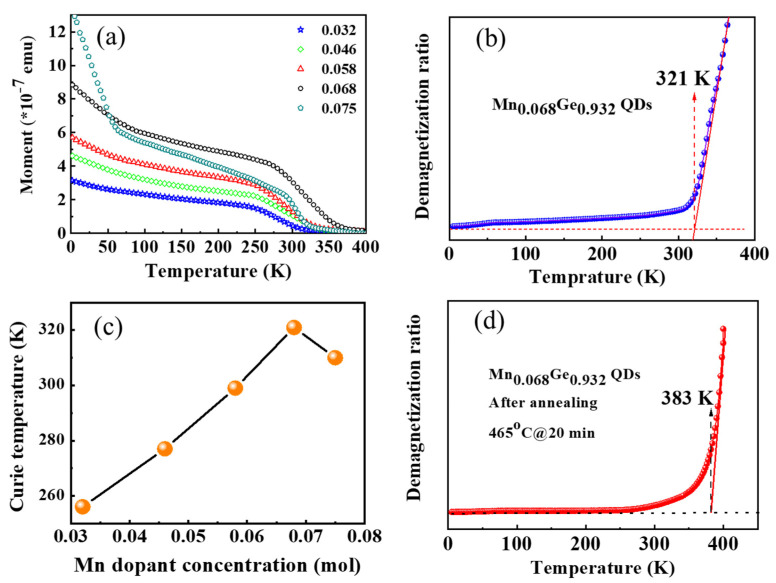
(**a**) Temperature dependence of magnetization for the samples with different Mn dopant concentrations. The testing temperature increased from 3 K to 400 K. An external magnetic field of 200 Oe is applied parallel to the sample surface. (**b**) The demagnetization ratio as a function of temperature for Mn_0.068_Ge_0.932_ QDs sample. (**c**) Curie temperature dependence of Mn dopant concentration. (**d**) The demagnetization ratio as a function of temperature for Mn_0.068_Ge_0.932_ QDs sample after annealing.

Figure 8a shows the temperature dependence of magnetization for the samples with different Mn doping levels. Therein, the ferromagnetic signals in the Mn_0.068_Ge_0.932_ QDs are most striking. Only one transition temperature (ferromagnetic phase) was observed in these MnGe QDs with a low doping level (0.000–0.058). However, the magnetic moment below ~55 K increases remarkably with further increasing the Mn doping level. Another transition temperature (paramagnetic phase) appears at ~55 K in the Mn_0.075_Ge_0.925_ QDs. Similar moment evolution of the Mn_0.075_Ge_0.925_ QDs was observed in MBE-grown counterparts [11]. The appearance of a paramagnetic phase below 55 K can be attributed to Mn oxides or Mn/Ge/Si oxides on the island surface [3]. This suggests that the Mn oxides or Mn/Ge/Si oxides are easy to form at higher Mn doping levels due to the existence of excess Mn atoms.

The *T_C_* values of these MnGe QDs can be determined generally by fitting the temperature-dependent demagnetization ratio with the Curie–Weiss equation [3,11,41]. Results indicate that the maximal *T_C_* value of 321 K is obtained in Mn_0.068_Ge_0.932_ QDs, as shown in Figure 8b,c, respectively. The Curie temperature of the IBCS-grown samples does not increase monotonically with the increase in Mn doping levels, but decreases when as Mn content is increased to 0.075. Hole-mediated effects generally dominate the ferromagnetism of the Mn-doped Ge alloy materials [3,11,14,42,43,44]. The localized Mn magnetic moments can be aligned to one direction by p-d exchange coupling between Mn ions and holes. Due to the stronger quantum confinement effect, the higher hole concentration confined in MnGe QDs can enhance the exchange coupling interaction and lead to the increase in the Curie temperature [6,45,46]. The exchange coupling interaction is enhanced with the increase in the Mn doping level until the formation of intermetallic precipitate at a relatively high Mn doping level. The formation of Mn_5_Ge_3-y_Si_y_ precipitates in Mn_0.075_Ge_0.925_ QDs sample has been well confirmed by XPS spectra. The Curie temperature of Mn_5_Ge_3-y_Si_y_ precipitate is definite, such as Mn_5_Ge_3_ and Mn_5_GeSi_2_ precipitate is 296 K and 225 K, respectively [15,47,48,49], which are lower than that of Mn_0.068_Ge_0.932_ QDs. Therefore, the existence of intermetallic precipitate depresses the ferromagnetic order in QDs and the further increase in the Curie temperature in the Mn_0.075_Ge_0.925_ QDs sample.

Both a residual strain and a higher Ge composition in QDs can benefit the formations of ferromagnetic phase Mn-doped Ge QDs. As shown in Figure 5, the Raman peak of the Si-Ge bond does not present until the Mn doping level is higher than 0.058, which suggests that Ge composition in the IBCS-grown MnGe QDs is higher than that in MBE-grown QDs. Furthermore, it is proposed that a strong residual strain is still left in undoped Ge QDs fabricated by using the IBSD [22,50]. These results suggest that IBCS is beneficial to forming Mn-doped Ge QDs with a good ferromagnetic phase. Although the maximal Curie temperature of MnGe QDs in this work does not reach 400 K, which is the highest Curie temperature of MnGe QDs reported in the literature [42], the overall *T_C_* values of the IBCS-grown MnGe QDs are superior to most of those with similar Mn doping levels grown by MBE [3,15]. To further enhance the ferromagnetism of MnGe QDs grown by IBCS, rapid thermal annealing for the Mn_0.068_Ge_0.932_ QDs samples were subjected to rapid thermal annealing at 465 °C for 20 min in a pure N_2_ environment. As shown in Figure 8d, the *T_C_* value of this sample increased from 321 K to 383 K after the post-annealing treatment; this suggested that a part of the defects in MnGe QDs were repaired during the post-annealing process; the FM order is well enhanced, correspondingly. However, more work on annealing is needed to reveal the ferromagnetic modulation of the MnGe QDs.

## 4. Conclusions

The Mn doping level dependent microstructure and ferromagnetism of Mn_x_Ge_1−x_/Si QDs were systematically investigated using ion beam co-sputtering technology. The ripening growth of Mn_x_Ge_1−x_ QDs, driven by self-assembly via the Stranski–Krastanov (SK) growth mode, was identified to result in a size increase and a density decrease in the Mn_x_Ge_1−x_ island with an increase in the Mn doping level. The distribution behavior of Mn atoms in QDs and Si buffer layers are well elucidated, based on the observation of the surface-enhanced Raman scattering effects. An optimal Mn doping level of 0.068 created the highest Curie temperature of MnGe QDs obtained in the investigated doping range; further increasing the Mn doping level to 0.075 decreased the Curie temperature, probably due to the formation of intermetallic precipitates in MnGe QDs. The steady enhancement of ferromagnetism has been demonstrated in the post-annealed Mn_0.068_Ge_0.932_ QDs sample by increasing the Curie temperature from 321 K to 383 K. Our results indicate that ion beam co-sputtering can be an effective alternative method for fabricating ferromagnetic nanomaterials. The highly ferromagnetic performance of Mn_x_Ge_1−x_ QDs confirmed in this work is conducive to accelerating the realization of the excellent engineering of full Si-based spintronic and micro-electronic integration**.**

## Data Availability

The data presented in this study are available in this article.
